# Predicting Spatial Variations in Soil Nutrients with Hyperspectral Remote Sensing at Regional Scale

**DOI:** 10.3390/s18093086

**Published:** 2018-09-13

**Authors:** Ying-Qiang Song, Xin Zhao, Hui-Yue Su, Bo Li, Yue-Ming Hu, Xue-Sen Cui

**Affiliations:** 1 College of Natural Resources and Environment, South China Agricultural University, Guangzhou 510642, China; yingq_s@stu.scau.edu.cn (Y.-Q.S.); xinzhao@stu.scau.edu.cn (X.Z.); huiyuesu@163.com (H.-Y.S.); bli@cse.ust.hk (B.L.); xuesen_scau@163.com (X.-S.C.); 2 Guangdong Province Key Laboratory for Land Use and Consolidation, Guangzhou 510642, China; 3 Guangdong Province Engineering Research Center for Land Information Technology, Guangzhou 510642, China; 4 Key Laboratory of Construction Land Transformation, Ministry of Land and Resources, Guangzhou 510642, China; 5 College of Agriculture and Animal Husbandry, Qinghai University, Xining 810016, China

**Keywords:** soil nutrients, hyperspectral remote sensing, artificial neural network, spatial variation

## Abstract

Rapid acquisition of the spatial distribution of soil nutrients holds great implications for farmland soil productivity safety, food security and agricultural management. To this end, we collected 1297 soil samples and measured the content of soil total nitrogen (TN), soil available phosphorus (AP) and soil available potassium (AK) in Zengcheng, north of the Pearl River Delta, China. Hyperspectral remote sensing images (115 bands) of the Chinese Environmental 1A satellite were used as auxiliary variables and dimensionality reduction was performed using Pearson correlation analysis and principal component analysis. The TN, AP and AK of soil were predicted in the study area based on auxiliary variables after dimensionality reduction, along with stepwise linear regression (SLR), support vector machine (SVM), random forest (RF) and back-propagation neural network (BPNN) models; 324 independent points were used to verify the predictive performance. The BPNN model, which demonstrated the best predictive accuracy among all methods, combined ordinary kriging (OK) with mapping the spatial variations of soil nutrients. Results show that the BPNN model with double hidden layers had better predictive accuracy for soil TN (root mean square error (*RMSE*) = 0.409 mg kg^−1^, *R*^2^ = 44.24%), soil AP (*RMSE* = 40.808 mg kg^−1^, *R*^2^ = 42.91%) and soil AK (*RMSE* = 67.464 mg kg^−1^, *R*^2^ = 48.53%) compared with the SLR, SVM and RF models. The back propagation neural network-ordinary kriging (BPNNOK) model showed the best predictive results of soil TN (*RMSE* = 0.292 mg kg^−1^, *R*^2^ = 68.51%), soil AP (*RMSE* = 29.62 mg kg^−1^, *R*^2^ = 69.30%) and soil AK (*RMSE* = 49.67 mg kg^−1^ and *R*^2^ = 70.55%), indicating the best fitting ability between hyperspectral remote sensing bands and soil nutrients. According to the spatial mapping results of the BPNNOK model, concentrations of soil TN (north-central), soil AP (central and southwest) and soil AK (central and southeast) were respectively higher in the study area. The most important bands (464–517 nm) for soil TN (b10, b14, b20 and b21), soil AP (b3, b19 and b22) and soil AK (b4, b11, b12 and b25) exhibited the best response and sensitivity according to the SLR, SVM, RF and BPNN models. It was concluded that the application of hyperspectral images (visible-near-infrared data) with BPNNOK model was found to be an efficient method for mapping and monitoring soil nutrients at the regional scale.

## 1. Introduction

As definitive indicators of soil fertility, soil nutrients play a pivotal role in agricultural productivity, food security and agro-ecological sustainable development [1]. The nitrogen, phosphorus and soil potassium contents of soil comprise the most crucial nutrients because they are closely related to nutrient cycling for crop growth and fertilizer application in human activities [2,3,4,5,6]. Applying variables to predict soil nutrients is a key means of clarifying their spatial variations. Variables such as topography, climate, vegetation and remote sensing can exhibit individual similarities in a single sample between environment parameters and soil nutrients and explain the gradual change difference in continuous space for their linear and nonlinear relationships. Therefore, faster and more accurate prediction of soil nutrients is essential to reducing soil nutrient loss and improving agricultural fertilization management in soil.

The spatial distribution of soil nutrients in farmland typing depends on field sampling and laboratory analysis, which are inefficient and time-consuming. Owing to their high efficiency and low cost, multispectral remote sensing and environmental factors have come to be used as auxiliary variables and in combination with different models to predict soil properties. Common soil properties include soil moisture [7], organic carbon [8], organic matter [9,10], clay [11], heavy metals [12,13] and soil nutrients [14,15]. Predictive techniques for soil nutrients can be classified into two categories. The first is linear prediction methods, which refers to the linear mathematical relationship between auxiliary variables (e.g., multispectral bands and environmental factors) and soil total nitrogen (TN), soil available phosphorus (AP) and soil available potassium (AK) to explain the variability and multi-order derivative relationship among soil nutrients. For example, due to a simple model structure and ease of use, multiple linear regression models (MLR) and partial least squares regression (PLSR) combined with multispectral remote sensing variables have been used to predict soil nutrients [16,17,18]. These models can explain the linear fitting characteristics between different wavelengths and soil nutrients to a certain extent. However, correlations between multispectral remote sensing band variables and soil nutrients are rarely linear in nature.

Because multi-spectral remote sensing bands are indirect and complex in terms of capturing changes in soil nutrient contents, the second prediction technique includes non-linear prediction methods. To overcome the deficiencies of linear prediction models that have difficulty interpreting non-linear characteristics between multispectral variables and soil nutrients, many machine learning models have been used to predict soil properties. For example, extreme learning machine (ELM), support vector machine (SVM) and random forest (RF) models have been employed in land surface mapping, image classification, plant properties mapping and soil properties mapping [19,20,21,22,23]. Compared with linear prediction, non-linear prediction has higher accuracy and more explanatory power regarding spectral changes in soil nutrients.

However, multispectral remote sensing variables possess a major disadvantage, namely discontinuous bands (i.e., wavelength spaces are large and the number of bands in near-infrared spectrum are few). The adsorption mode of soil nutrients in farmland can be divided into two types: unstable external adsorption, which mostly occurs on the surface of soil organic matter (SOM); and relatively stable internal adsorption, which are usually adsorbed on the soil minerals. However, the spectral absorption of soil nutrients varies greatly at different wavelengths. Multispectral bands are fewer in number and narrower in wavelength range, which renders continuous spectral monitoring and evaluation of the complex variability in soil nutrients challenging at the regional scale. Hyperspectral remote sensing variables can capture weak spectral changes in soil nutrients due to their sufficient spectral resolution. These hyperspectral bands have a higher response and sensitivity to soil nutrients (e.g., TN, AP and AK) compared with multispectral remote sensing. Detailed hyperspectral bands also reflect the physical mechanisms of different linear and non-linear models in terms of their complex relationships with soil nutrient contents. Hybrid kriging models have recently been widely used to improve mapping accuracy by combining non-spatial algorithms with geostatistical interpolation algorithms [24,25,26,27]; however, incorporating hyperspectral remote sensing images and the hybrid kriging model has not yet been studied in the predicting soil nutrient contents.

However, there is a demand to quantify the effects of hyperspectral remote sensing images in improving prediction via linear and non-linear methods such as SLR, SVM, RF and ANN models. Further research has not yet proven whether the hybrid kriging model is more suitable in evaluating the variability and spatial mapping of soil nutrients. The aim of this study is to compare the ability of different linear, non-linear machine learning and hybrid kriging models in predicting soil nutrient contents (TN, AP and AK) content using hyperspectral remote sensing images as auxiliary variables.

## 2. Materials and Methods

### 2.1. Study Area

**Figure 1:**
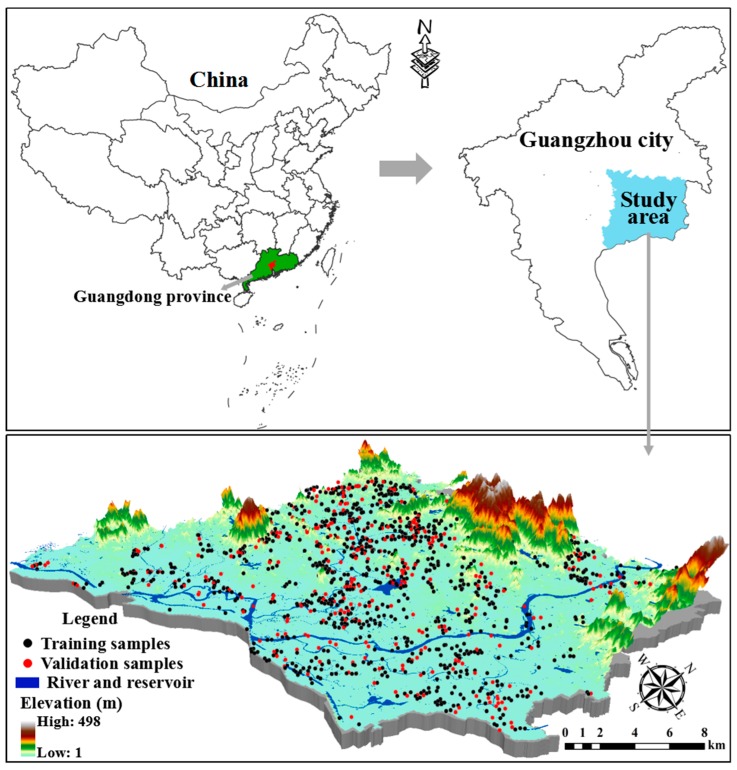
Location of study area in the northeast of Pearl River Delta (PRD) and distribution of the training and validation sites.

The study area was carried out in Zengcheng (23°5′ to 23°37′ N and 113°29′ to 114°0′ E), which is located in the northeast of Pearl River Delta (PRD), China (Figure 1). The climate of the study area is best defined as a south subtropical oceanic monsoon climate, with an annual average temperature of 21.6 °C, an annual average sunshine of 1785.5 h, an annual average relative humidity of 79% and an annual average precipitation of 1994.5 mm. Because of the warm and humid climate, vegetables and food crops can be grown year-round. The topography types are characterized by plain and hills with a maximum elevation of 1084.3 m. According to Chinese soil classification, soil types in farmland in the study area are classified as red soils and latosolic red soils. The study area is also a typical agricultural zone in the PRD and the traditional planting structure is that of a paddy farming system producing crops and vegetables.

### 2.2. Field Sampling and Chemical Analysis

In order to reduce the interference of soil moisture, cloud cover and crops on the hyperspectral information obtained by the remote sensor, we chose to collect samples in the driest months (October–December 2008), to ensure bare soil in farmland after crops had been harvested. Using the basic grid partition method, five soil samples were randomly collected in five corners of an “X” shape. According to the actual size of fields, the area representing “X” shape is from 100 m^2^ to 500 m^2^ and mixed samples can avoid the content mutation of soil nutrients by single sample. The regularization problems which due to the average over the point spread function (PSF) in remote sensing images. The unit of regularization (i.e., resolution) in remote sensing image is very important for prediction results of soil properties. Regularization effects are capable of affecting the nonlinear structures embedded in high-dimensional space, with the great potential for computational efficacy, unmixing accuracy and robustness to noise in hyperspectral images [28,29].

After the samples were mixed thoroughly, we collected 1 kg of each sample; 1297 topsoil (0–20 cm) samples were collected in this study (Figure 1). The GPS data of all samples were recorded before samples were air-dried naturally at room temperature. After removing plant residues and stones, all samples were passed through a 100-mesh nylon sieve (0.2 mm). The determination of soil total nitrogen (TN) was measured by the method described by Walkley and Black [30]; the soil available phosphorus (AP) was extracted by 0.5 mol L^−1^ NaHCO_3_ and then measured via the Mo-Sb colorimetric method; and soil available potassium (AK) was extracted by 1 mol L^−1^ NH_4_-OAc with 1:5 weight-to-volume ratio and then measured by flame photometry [31].

### 2.3. Hyperspectral Auxiliary Variables

In order to guarantee the accurate correspondence between the remote sensing image and soil samples, we selected the hyperspectral image from October 2008, which had the most recent field sampling time and the least cloud cover. The hyperspectral images were obtained from the Chinese Environment 1A satellite, available from the China Centre for Resources Satellite Data and Application Server (http://www.rscloudmart.com/). The bands of Environment 1A have the spectral sampling distance between bands of 4.32 nm (spectral range: 450–950 nm) and consisted of 115 bands. Environment 1A also had a pixel size of 100 m × 100 m, sufficient for monitoring the spatial distribution of soil nutrients at the regional scale. All spectral bands were geo-referenced using the same coordinate system as in soil nutrient sampling. Atmospheric correction, fast line-of-sight atmospheric analysis of spectral hypercubes and decomposition of mixed pixels were used to eliminate sensor error such as from the atmosphere, sun angle and hybrid spectra of multiple objects in the hyperspectral image. Spectral pre-processing was performed using analyst tools in ENVI 5.2 (Boulder, CO, USA).

### 2.4. Correlation Analysis and Principal Component Analysis

The high dimensionality of hyperspectral bands results in high information redundancy and auto-correlation. Screening effective variables from hyperspectral bands improves model efficiency and optimizes the model structure. The Pearson correlation analysis and principal component analysis (PCA) were used to reduce the dimensions of hyperspectral bands [12,14]. On the one hand, auto-correlated bands were removed among 115 hyperspectral bands using Pearson correlation analysis. On the other hand, the PCA was applied to address the multi-collinearity of auxiliary variables, as it is an effective method to reduce variable dimensions and hence obtain the optimal principal components (PCs). PCs were then used as input data for subsequent predictive models. The SPSS 22.0 (IBM Corp., Armonk, NY, USA, 2013) was used to implement the Pearson correlation analysis and PCA.

### 2.5. Non-Spatial Prediction Models

The stepwise linear regression (SLR) model is often used to assess the linear relationship between multiple independent variables and it can be used for variable screening and avoided collinearity for soil nutrient prediction [16]. Stepwise regression can also be used for variable screening. SLR can remove the weakly significant variables while retaining those with high contribution rate. In this study, the SLR model was used to identify the optimal combination of input PCs with targets. The linear fitting relationships between hyperspectral auxiliary variables and soil nutrients were also extracted. The SLR model was performed in MATLAB 2013b software. The formula of SLR model (Equation (1)) is defined as follows [32]:

(1) $$y=b+a_{1}x_{1}+\cdots +a_{k}x_{k},$$

where y is the estimation value of SLR for soil nutrients, *b* is a regression constant, $a_{1},\ldots ,a_{k}$ are regression coefficients and $x_{1},\;\ldots ,x_{k}$ are the input PCs converted from hyperspectral variables.

The support vector machine (SVM) model is a supervised learning method that was used to solve the regression problem in this study. The SVM method can identify the separating optimal hyperplane in multi-dimensional spatial data and seeks to minimize the error of all training samples. The SVM model overcomes the limitation of neural networks, which tend to rely on local optimal solutions [33]. Therefore, the SVM model is well suited to soil nutrient prediction with multi-dimensional variables.

Given a dataset with *N* samples, $\left\{\left(x_{1},y_{1}),\ldots ,(x_{i},y_{i}\right)\right\},\;i=1,\ldots ,N$ (where $x_{i}\in R^{n}$ is the input vector, $y_{i}\in R^{1}$ is a target output and *N* is the number of data points), the standard form of SVM [34] is as follows:

(2) $$\begin{array}{ll} \underset{\omega ,b,\xi ,\xi^{*}}{\min} & \frac{1}{2}\omega^{T}\omega +C\sum_{i=1}^{N}\xi_{i}+C\sum_{i=1}^{N}\xi_{i}^{*}\\ \;s.t. & \omega^{T}\varphi (x_{i})+b-y_{i}\leq \varepsilon +\xi_{i}\\ & y_{i}-\omega^{T}\varphi (x_{i})-b\leq \varepsilon +\xi_{i}^{*}\\ & \xi_{i},\xi_{i}^{*}\geq 0,i=1,\ldots ,N \end{array}$$

where $\varphi (x_{i})$ maps the input space to the feature space, ω and b are optimized coefficients during the training phase, ε is the insensitive loss function, *C* is the regularized constant and $\xi_{i}$ and $\xi_{i}^{*}$ are two positive slack variables. The dual form is

(3) $$\begin{array}{ll} \underset{\alpha ,\alpha^{*}}{\min} & \frac{1}{2}(\alpha -\alpha^{*}{)}^{T}Q(\alpha -\alpha^{*})+\varepsilon \sum_{i=1}^{N}\left(\alpha_{i}+\alpha_{i}^{*}\right)+\sum_{i=1}^{N}y_{i}(\alpha_{i}-\alpha_{i}^{*})\\ \;s.t. & \sum_{i=1}^{N}\left(\alpha_{i}-\alpha_{i}^{*}\right)=0,\alpha_{i}\geq 0,\alpha_{i}^{*}\leq C,i=1,\ldots ,N \end{array}$$

where $\alpha_{i}$ and $\alpha_{i}^{*}$ are non-negative Lagrangian multipliers; therefore, the regression function can be given as

(4) $$\sum_{i=1}^{N}\left(\alpha_{i}^{*}-\alpha_{i}\right)K(x_{i},x)+b,$$

where $K(x_{i},x)$ is the kernel function. In this study, the radial basis function (RBF) was used as the kernel function. The libsvm package [35] was imported into the MATLAB 2013b software to construct the SVM model and a multi-dimensional fitting relationship between soil nutrients content and the input PCs was established [36,37,38,39]. The SVM model parameters set as follows: the type of SVM was epsilon support vector regression (SVR); the meshgrid function was used to find optimum parameters, which including the parameter C of epsilon-SVR and gamma in kernel function; the epsilon in loss function of epsilon-SVR was 0.01 [35].

**Figure 2:**
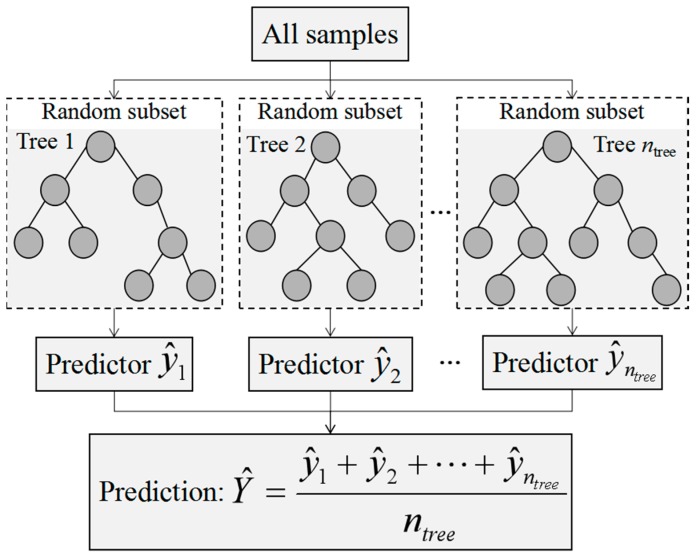
The main structure of random forest algorithm.

The random forest (RF) model was developed from the decision tree models using ensemble learning methods [40]. The RF model is an algorithm as well as an idea of combination and integration. The main structure of the RF algorithm is depicted in Figure 2. First, the bootstrap method was used to randomly form *n*_tree_ samples from the original training dataset from which multiple classification regression trees were constructed (${\hat{y}}_{1},{\hat{y}}_{2},\ldots ,{\hat{y}}_{n_{tree}}$). Then, voting rules were used to divide the optimal tree nodes and the average values of decision trees (i.e., $\hat{Y}$) with the most output were taken as the prediction results [41,42]. The RF model solves the overgrowth phenomenon of decision trees with non-equilibrium sample fitting and does not need to be pruned. In this study, the number of decision trees was 1000 and the number of variables split by each decision tree model was 2. The RF model was carried out in MATLAB 2013b.

The artificial neural networks (ANNs) comprise a classic nonlinear prediction model. ANNs can be used to model complex nonlinear relationships with limited discontinuous points between hyperspectral auxiliary variables and soil nutrients. Based on self-adjustment of internal control parameters in the human brain, the ANN model includes three layers (an input layer, hidden layer and output layer) [43,44]. A back-propagation neural network (BPNN), given its structural simplicity and robustness in simulation [45], was applied to estimate soil nutrient contents in the study. The BPNN model can also incorporates the need to adjust activation functions (i.e., the sigmoid function) (Equation (7)), number of neurons (*n*) and weights (i.e., input weights $\omega_{ij}$ and output weights $\omega_{jk}$). The input of the hidden layer (Equation (5)) and that of the output layer (Equation (6)) are calculated as follows [46]:

(5) $$H_{j}=\sum_{i=1}^{n}\omega_{ij}x_{i}+\beta_{j},j=1,2,\ldots ,n,$$

(6) $$V_{k}=\sum_{j=1}^{m}\omega_{jk}s_{j}+\beta_{k},k=1,2,\ldots ,m,$$

where $z(x_{i})$ and $V_{k}$ are the inputs of the hidden layer and the output layer, respectively; N(h) is the variable of the $\lambda_{i}$th input node; and $\beta_{j}$ and $\beta_{k}$ are the bias values of the hidden layer and the output layer, respectively. The sigmoid function $s_{j}$ is applied as follows:

(7) $$s_{j}=f(x)=\frac{1}{1+\exp(-x)},$$

In order to fully exploit the nonlinear characteristics between hyperspectral variables and soil nutrients, a BPNN model with two hidden layers was constructed in this study which to ensure model stability. The BPNN model was performed in MATLAB 2013b.

### 2.6. Hybrid Kriging Method

The hybrid kriging method (i.e., artificial neural network–ordinary kriging (ANNOK)) integrated a non-spatial method (the BPNN model) and spatial interpolation approach of ordinary kriging. First, according to the established BPNN model between input PCs and targets, the residuals (Equation (8)) of BPNN were calculated by their estimated and measured values. Second, the semivariogram (Equation (10)) [47] and ordinary kriging (Equation (9)) of BPNN residuals were calculated. Finally, the estimated values of the BPNN method and the ordinary kriging values of the BPNN residuals were summed (Equation (11)).

(8) $$r(x_{i})=z(x_{i})-{z^{\prime }}_{BPNN}(x_{i}),$$

(9) $$\hat{z}(x_{i})=\sum_{i=1}^{n}\lambda_{i}r(x_{i}),$$

(10) $$\hat{\gamma }(h)=\frac{1}{2N(h)}\sum_{i=1}^{N(h)}{\left[r(x_{i})-r(x_{i}+h)\right]}^{\;2},$$

(11) $${\hat{z}}_{sum}(x_{i})={z^{\prime }}_{BPNN}(x_{i})+\hat{z}(x_{i}),$$

where $r(x_{i})$ is the residual value of the BPNN model at sampling site $x_{i}$; $z(x_{i})$ and ${z^{\prime }}_{BPNN}(x_{i})$ are the measured soil nutrient values and the BPNN model estimate, respectively; $\hat{z}(x_{i})$ denotes the estimated BPNN residuals at a site $x_{i}$ with ordinary kriging; n is the number of soil samples; $\lambda_{i}$ is the optimal weight; $\hat{\gamma }(h)$ and N(h) denote the experimental semivariogram and number of pairs of sampling sites separated by h (a lag distance between $r(x_{i})$ and $r(x_{i}+h)$); and ${\hat{Z}}_{sum}(x_{i})$ is the final estimated value of soil nutrient contents by back propagation neural network-ordinary kriging (BPNNOK). The ArcGIS 10.3 was used to implement spatial interpolation and geostatistical calculations.

### 2.7. Validation of Predictive Performance

An independent validation dataset (324 samples, 25% percentage of the total dataset) was randomly extracted using the “create subset” function in ArcGIS 10.3. The validation set did not participate in the model training as independent verification. The mean absolute error (*MAE*) (Equation (12)), root mean square error (*RMSE*) (Equation (13)), coefficient of determination (*R*^2^) (Equation (14)) and ratio of performance to deviation (*RPD*) (Equation (15)) [48] were calculated to assess the predictive accuracy of soil nutrients.

(12) $$MAE=\frac{1}{n}\sum_{i=1}^{n}\left|z^{\prime }(x_{i})-z(x_{i})\right|,$$

(13) $$RMSE=\sqrt{\frac{1}{n}\sum_{i=1}^{n}{\left(z^{\prime }(x_{i})-z(x_{i})\right)}^{2}},$$

(14) $$R^{2}={\left[\frac{\sum_{i=1}^{n}\left(z(x_{i})-\bar{z(x_{i})}\right)\left(z^{\prime }(x_{i})-\bar{z^{\prime }(x_{i})}\right)}{\sqrt{\sum_{i=1}^{n}{\left(z(x_{i})-\bar{z(x_{i})}\right)}^{2}{\left(z^{\prime }(x_{i})-\bar{z^{\prime }(x_{i})}\right)}^{2}}}\right]}^{2},$$

(15) $$\mathrm{RPD}=\frac{STD}{RMSE},$$

where $z^{\prime }(x_{i})$ is the estimated value per the SLR, SVM, RF and BPNN models; and $\bar{z^{\prime }(x_{i})}$ is the stationary mean of $z^{\prime }(x_{i})$. *STD* is the standard deviation of soil nutrient measurement (mg kg^−1^), where a higher *RPD* value indicates greater accuracy for the quality of prediction models across three classes: the lowest predictive performance (*RPD* ≤ 1.4), fairly acceptable predictive performance (1.4 < *RPD* < 2) and accurate predictive performance (*RPD* ≥ 2) [49,50].

## 3. Results

### 3.1. Descriptive Statistics for Soil Nutrients

Descriptive statistical analysis of soil nutrients was performed based on the training set and validation set (Table 1). In general, the statistical values of training set and validation set were highly similar. For the training set, the concentrations of soil total nitrogen (TN), soil available phosphorus (AP) and soil available potassium (AK) ranged from 0.12 mg kg^−1^ to 3.04 mg kg^−1^, 2.2 mg kg^−1^ to 261.6 mg kg^−1^ and 7 mg kg^−1^ to 491 mg kg^−1^ with a mean value of 1.18 mg kg^−1^, 75.54 mg kg^−1^ and 103.16 mg kg^−1^, respectively. Concentrations for the validation set ranged from 0.15 mg kg^−1^ to 2.98 mg kg^−1^, 2.4 mg kg^−1^ to 257.5 mg kg^−1^ and 6 mg kg^−1^ to 486 mg kg^−1^, with mean values of 1.22 mg kg^−1^, 74.81 mg kg^−1^ and 100.55 mg kg^−1^, respectively. AP and AK values each demonstrated skewness and kurtosis greater than 0. The Kolmogorov-Smirnov (K-S) test of TN in the validation set was larger than 0.05, whereas other soil nutrients indicated a non-normal distribution in the training set and validation set. Furthermore, the coefficients of variation (CV) of soil TN, soil AP and soil AK in the training dataset were 45.15%, 71.28% and 88.03%, respectively (CV > 35% is considered highly variable) [51]. These values in the validation dataset were 43.46%, 71.54% and 88.68%, respectively. The high CV of soil nutrients was likely due to the fragmentation of farmland distribution and the strong interference of fertility management in the study area. In general, the raw data were appropriate for the SLR, SVM, RF and BPNN models because these models did not exhibit interference from the non-normal distribution and sampling density of data [19,32].

### 3.2. Correlation Analysis and Principal Component Analysis

**Figure 3:**
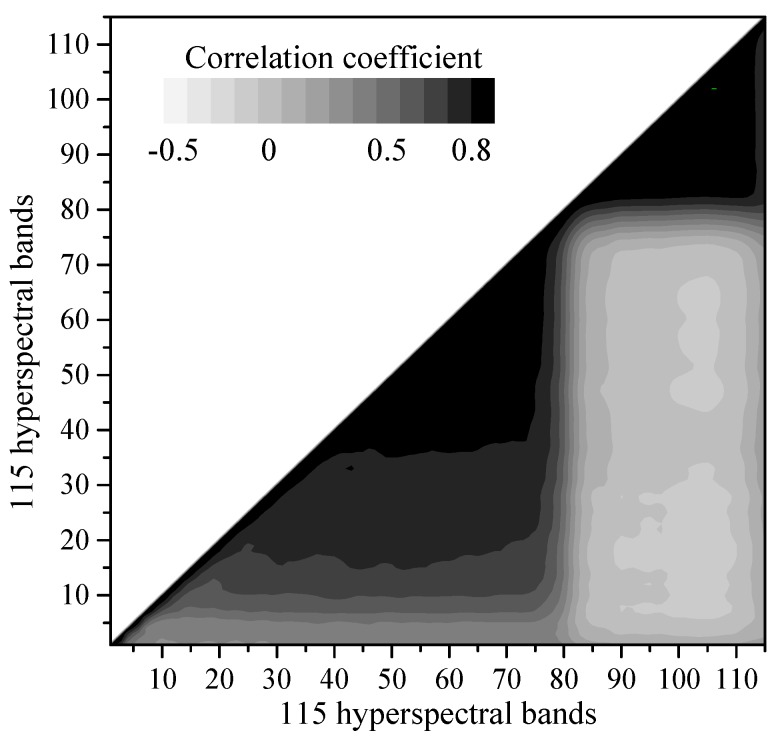
The Pearson correlation analysis among 115 hyperspectral bands.

Figure 3 displays the Pearson correlation analysis among 115 hyperspectral bands. The hyperspectral variables within the 21–77 bands (527–694 nm) and 81–115 bands (734–930 nm) demonstrated significant positive correlations (*p* < 0.01). Due to their high autocorrelation and information redundancy, variables within the 22–72 bands and 83–115 bands were eliminated to reduce the dimensions and simplify and the model structure. In order to remove the multi-collinearity in hyperspectral variables, the PCA was performed based on the excluded hyperspectral auxiliary variables. After removing auto correlated bands, the PCA of the hyperspectral variables revealed that the eigenvalues of the first five PCs were greater than 1 and the cumulative contribution rate reached 90.46% (Table 2). Compared with other PCs, PC1 (62.45%) loaded heavily on bands 4–21 and bands 73–82, whereas other PCs contributed less to the hyperspectral bands.

In addition, Pearson correlation analysis was performed on the extracted five PCs with soil TN, soil AP and soil AK, respectively. Table 2 shows that different soil nutrients responded differently to the PCs. Results revealed a significant positive correlation between soil TN and PC3 (r = 0.079, *p* < 0.01) and PC4 (r = 0.079, *p* < 0.01) and a significant negative correlation with PC1 (r = −0.056, *p* < 0.05). Soil AP had a significant positive correlation with PC1 (r = 0.131, *p* < 0.01) and PC2 (r = 0.145, *p* < 0.01) and soil AK was significantly positively correlated with PC1 (r = 0.126, *p* < 0.01) and PC2 (r = 0.147, *p* < 0.01). These findings suggest that variability in the relationship between hyperspectral variables and soil TN was much higher than that between soil AK and soil AP. According to the 2008 Guangzhou Statistical Yearbook, the amount of nitrogen applied in the study area (1.11 × 10^4^ t) was far more than that of phosphate fertilizer (0.63 × 10^4^ t) and potash fertilizer (0.54 × 10^4^ t). A possible reason for the low correlation between hyperspectral variables and soil TN is that the elevation undulations in the study area were too large and agricultural activities were intense. The first five PCs were used as input data for subsequent predictive models.

### 3.3. Prediction of Soil Nutrients by SLR, SVM, RF and BPNN Models

SLR, SVM, RF and BPNN models were respectively constructed based on the five PCs derived from Pearson correlation analysis and PCA. The SLR model was used to fit the prediction equation for soil TN (Equation (16)), soil AP (Equation (17)) and soil AK (Equation (18)) using input PCs drawn from the original hyperspectral variables. Table 3 shows the predictive accuracy in the validation dataset for soil TN (*RMSE* = 0.468 mg kg^−1^, *R*^2^ = 18.92%), soil AP (*RMSE* = 46.81 mg kg^−1^, *R*^2^ = 21.31%) and soil AK (*RMSE* = 80.57 mg kg^−1^, *R*^2^ = 24.69%). The results of the fitted SLR model could only explain the linear changes in soil nutrients. Among the five PCs, PC3 and PC4 were each positively and significantly correlated to soil TN, whereas PC1, PC2 and PC3 were more important for AP and AK. The fitting accuracy of soil TN by the SLR model was low, indicating that the variability of TN was higher as evidenced by its significant negative correlation with PC1.

SLR_TN_ = 1.217 − 0.044PC1 + 0.338PC3 + 0.765PC4, (16)

SLR_AP_ = 66.371 + 12.621PC1 + 61.14PC2 − 29.157PC3, (17)

SLR_AK_ = 89.258 + 20.704PC1 + 103.263PC2 − 48.294PC3, (18)

Among the radial basis, sigmoid, polynomial and linear function kernel functions of the SVM model, the SVM model constructed by the polynomial kernel functions had the highest predictive accuracy. The five PCs were used as input data to predict soil TN, soil AP and soil AK, respectively. As shown in Table 3, the fitting accuracy of the SVM model from high to low was soil AK (*RMSE* = 77.903 mg kg^−1^, *R*^2^ = 29.52%) > soil TN (*RMSE* = 0.448 mg kg^−1^, *R*^2^ = 27.00%) > soil AP (*RMSE* = 45.634 mg kg^−1^, *R*^2^ = 26.44%). Compared with the SLR model, the SVM model demonstrated improved the predictive accuracy for soil TN, soil AP and soil AK; however, the fitting ability for soil nutrient variability was unsatisfactory because the polynomial kernel function is better suited for problems in normalized training data, which failed to fully explain complex nonlinear relationships between the hyperspectral variables and soil nutrients.

For the RF model, the five PCs were used as input data to predict the soil TN, soil AP and soil AK. As listed in Table 4, the accuracy of the fitting results was soil TN (*RMSE* = 0.420 mg kg^−1^, *R*^2^ = 38.32%) > soil AK (*RMSE* = 72.972 mg kg^−1^, *R*^2^ = 35.12%) > soil AP (*RMSE* = 43.217 mg kg^−1^, *R*^2^ = 34.21%), indicating that the RF model had a better fitting ability for variability in soil TN. Furthermore, in the decision tree training process, the number of trees was set to different values (200, 500 and 1000, respectively). The fitting accuracy improved in line with the number of trees but the operation efficiency declined significantly. Compared with the SLR and SVM models, the RF model exhibited better predictive accuracy for soil nutrients and better reflected complex nonlinear relationships between the hyperspectral variables and soil nutrients.

Table 5 presents the network architecture and results of the BPNN model. For soil TN, soil AP and soil AK, the best network architecture (input layer, hidden layer and output layer) was 5-25-20-1, 5-20-15-1 and 5-20-15-1, respectively, according to the sigmoid kernel function. Compared with the prediction results of soil TN and soil AP, the BPNN model had the highest predictive accuracy for soil AK, implying that BPNN had a better fitting ability for the variability of soil AK. The BPNN model with a double hidden layer structure demonstrated better stability and generalization. By increasing the number of neurons in the hidden layer, the predictive accuracy was approximately 1 but resulted in overfitting.

**Figure 4:**
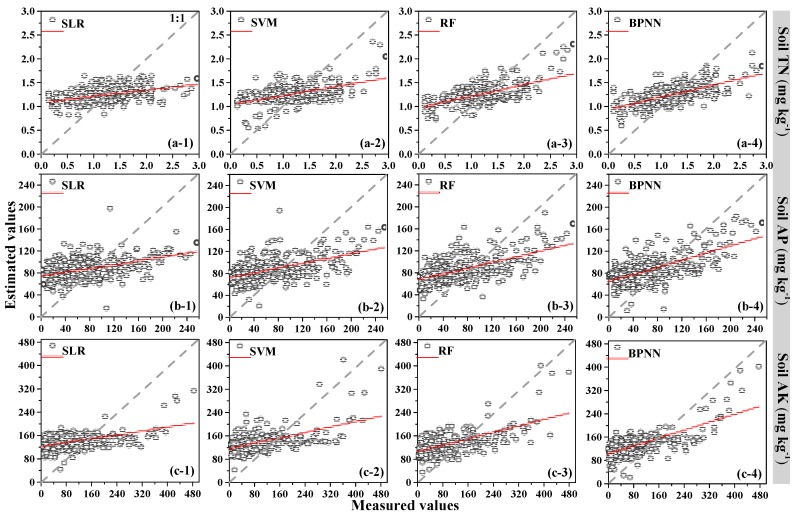
Observed versus estimated (**a**) soil TN; (**b**) soil AP; and (**c**) soil AK, using SLR, SVM, RF and BPNN models at the validation set.

Compared to all prediction models, the explanatory power of the soil TN from high to low was BPNN (44.24%) > RF (38.32 %) > SVM (27.00%) > SLR (18.92%); the explanatory power of the soil AP from high to low was: BPNN (42.91%) > RF (34.21%) > SVM (26.44%) > SLR (21.31%); and the explanatory power of soil AK was BPNN (48.53%) > RF (35.12%) > SVM (29.52%) > SLR (24.69%). Figure 4 shows the estimated values of the soil nutrient contents which were plotted against the measured values for validation sites. The scatter plots of the BPNN model for the soil TN, soil AP and soil AK had the better compactness and fewer outliers compared with the other models. The BPNN model essentially exhibited a more powerful nonlinear ability between hyperspectral auxiliary variables and soil nutrients to some extent; therefore, this model was selected to map the spatial distribution of soil nutrients with the ordinary kriging of residuals.

### 3.4. Spatial Prediction of Soil Nutrients Content by the BPNNOK Model

**Figure 5:**
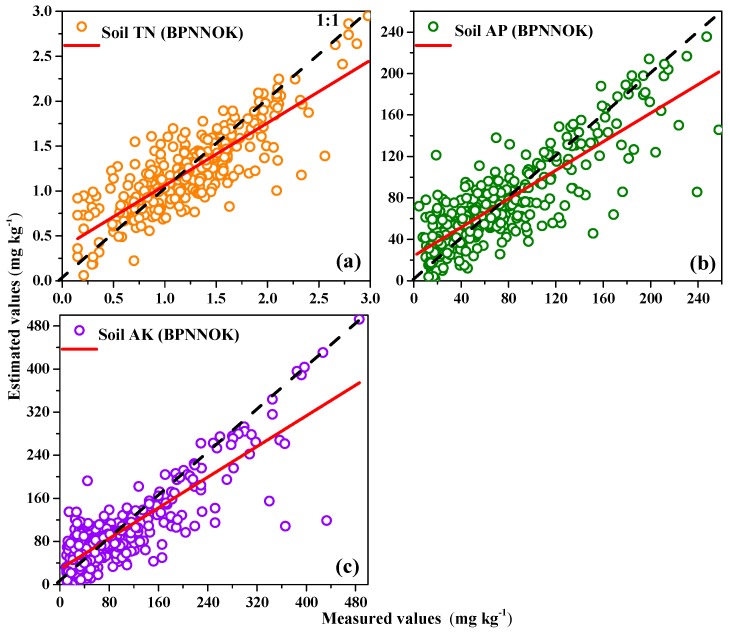
Measured values versus estimated values of (**a**) soil TN; (**b**) soil AP; and (**c**) soil AK, using BPNNOK model.

**Figure 6:**
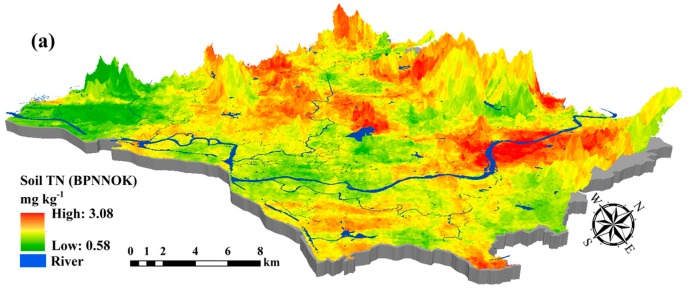

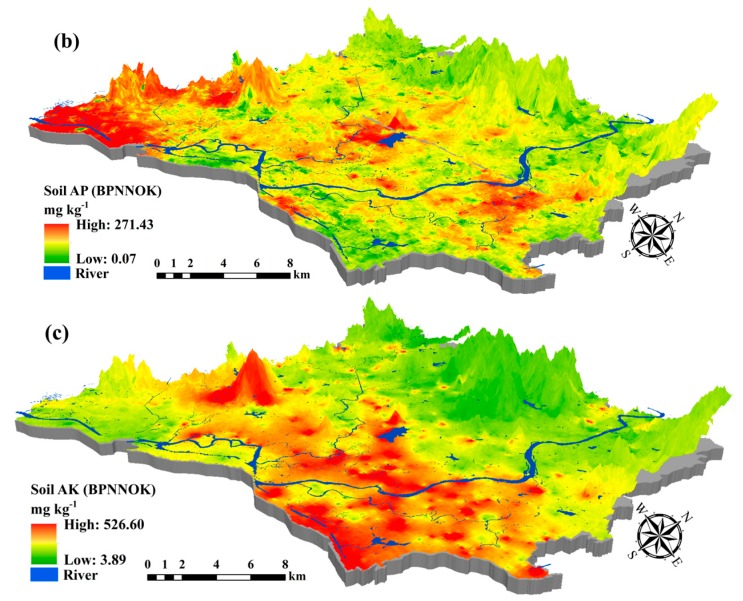
Predictive maps of (**a**) soil TN; (**b**) soil AP; and (**c**) soil AK using BPNNOK model in 100 m × 100 m resolution.

The Gaussian exponential models were selected based on the minimum nugget effect (NE) value among 11 function models during semivariogram modeling using ArcGIS10.3 software. The semivariogram parameters of BPNN residuals for the soil nutrient contents are listed in Table 6. The NE was used to describe the spatial variability and dependence of the residuals on the soil nutrient contents. The NE in this study showed that the ordinary kriging residuals of the BPNN model demonstrated high spatial dependence (NE > 75%) [52] for the soil TN (96.1%), soil AP (96.8%) and soil AK (86.5%). Compared to SVM, RF and BPNN, the OK residuals of the BPNN exhibited higher *RMSE* for soil TN (0.453 mg kg^−1^), soil AP (45.91 mg kg^−1^) and soil AK (79.56 mg kg^−1^), respectively. There are two reasons for the low accuracy of ordinary kriging residuals. On the one hand, residuals of the BPNN exhibited the weak spatial auto-correlation after removing the secondary predictors’ effects in comparison with raw dataset of soil nutrients; On the other hand, the ordinary kriging model was difficult to fit the weak nonlinear relationships between hyperspectral variables and soil nutrients in comparison with nonlinear machine learning models. Compared to soil AP and soil AK, the scatter plots of the BPNNOK model for the soil TN had the best compactness and the fewest outliers; additionally, the point distribution demonstrated homogeneity randomly around the 1:1 line (Figure 5). Because the BPNN model had the highest predictive accuracy among all models, the BPNNOK model was constructed according to Equation (11) and the spatial mapping of soil TN, soil AP and soil AK was respectively carried out using hyperspectral remote sensing images from the study area (Figure 6).

Compared with the SLR, SVM, RF and BPNN models, the BPNNOK had the highest predictive accuracy and the best fitting ability for the spatial variability of soil nutrients (Table 7). Figure 6 shows the main concentrations for soil TN (north-central), soil AP (central and southwest) and soil AK (central and southeast) in the study area were respectively higher, whereas soil TN (southwest), soil AP (north) and soil AK (north and southwest) were generally lower. The BPNNOK prediction results for all soil nutrients included spatial transitions and gradients, which conformed to the law of geography. However, the concentrations for soil TN (north), soil AP (southwest) and soil AK (midwest) were respectively high in high-elevation area. Potentially, the artificial terraced fields and reservoirs caused a gradient change in soil nutrients from the hilltop to the bottom. Additionally, no rivers exported soil nutrients, resulting in high concentrations in the high-elevation area. The prediction ranges of soil TN content calculated by BPNNOK were much closer to the measured values, whereas the upper limits of all soil nutrient contents were higher than the measured values in the validation set. In short, the BPNNOK model performed the best understanding in terms of the spatial variability of soil nutrients, especially regarding the fitting ability between hyperspectral variables and soil nutrient contents.

## 4. Discussion

### 4.1. Driving Bands Influencing Predictive Performance of Soil Nutrients

**Figure 7:**
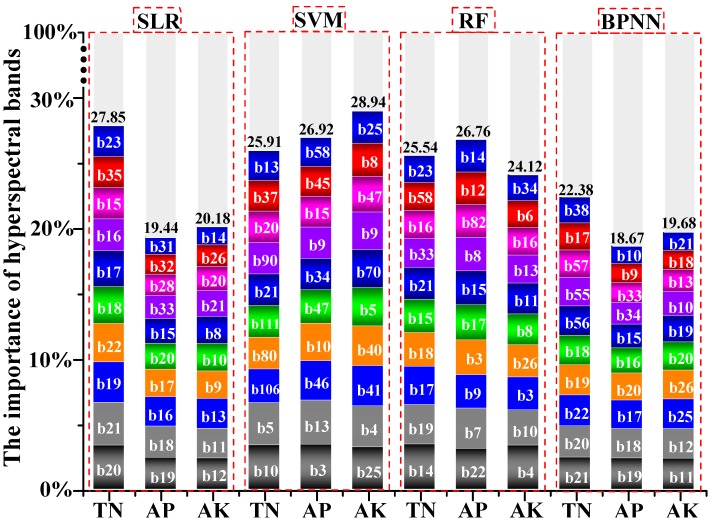
Driving force of hyperspectral bands for soil nutrients using SLR, SVM, RF and BPNN models.

The whole 115 bands of hyperspectral image were used to calculate bands’ importance in the prediction process for soil nutrients by the SLR, SVM, RF and BPNN models. The proportion of bands’ importance with respect to loss accuracy as part of total accuracy was also calculated (Figure 7). The first 10 important bands (464–773 nm) had a higher driving force for soil TN (average = 25.42%), soil AP (average = 22.95%) and soil AK (average = 23.23%) according to the SLR, SVM, RF and BPNN models. Furthermore, the most important bands (464–517 nm) for soil TN (b10, b14, b20 and b21), soil AP (b3, b19 and b22) and soil AK (b4, b11, b12 and b25) exhibited the best response and sensitivity for linear/non-linear fitting ability in the SLR, SVM, RF and BPNN models. However, due to traces of soil nutrients and strong disturbance from human activities, major spectral features could not be clarified [53]. Studies have shown that soil nutrients within the visible near infrared (VNIR) region (400–1000 nm) have the best effect. The application of the region of visible-near-infrared to shortwave-infrared region (VNIR-SWIR) is more expensive and accessible with a lower signal-to-noise ratio (SNR); however, the VNIR region is most widely used for predicting soil properties related to soil nutrients, such as soil biochar [54], soil organic matter [55,56,57] and others.

In this study, the driving performance of different bands to soil TN, soil AP and soil AK was also affected by elevation and human agricultural management. Elevation affects surface soil thickness (i.e., organic matter thickness) by influencing temperature and precipitation [58]. Soil thickness is closely related to the spectral absorption of soil nutrient contents. Human agricultural management, especially fertilization, plays a pivotal role in the seasonal exogenous input of soil nutrients, as well as corresponding spectral characteristics. Actually, environmental parameters such as topography, climate, vegetation and SWIR exhibited complex relationship with physical and chemical changes of soil properties [58,59]. It is significant for future research to predict the spatial variations in soil nutrients with multi-source environmental factors. In short, the application of hyperspectral imaging VNIR data (450–950 nm) in this study facilitated rapid, efficient mapping and monitoring of soil nutrients at the regional scale.

### 4.2. Predictive Performance of Soil Nutrients by Model Mechanism Analysis

The results of this paper reveal a weak linear relationship exists between soil nutrients and the hyperspectral variables and its nonlinear and multi-directional characteristics are significant. Therefore, the SLR model could hardly fit the complex relationship between soil nutrients and hyperspectral variables. The SVM model, which mapped the hyperplane of a multi-dimensional space to a two-dimensional plane, had low predictive accuracy. Because the types of hyperspectral variables selected in this study were limited to visible light components, the fitting capability of the SVM model was limited. The RF model also combined many trees to form a voting allocation mechanism, which is more appropriate for solving multivariate fitting problems. However, with an increase in the feature learning increments and number of trees, the computational efficiency of the RF model declined substantially. The multi-layer BPNN model used a complex weight calculation among hidden layers to improve the predictive accuracy and model stability and demonstrated a strong ability to fit non-linearity between hyperspectral variables and soil nutrients. In fact, there should be a linear and nonlinear relationship between hyperspectra and soil nutrients. Compared with a single linear or nonlinear model, the hybrid kriging model (BPNNOK) not only involves the prediction accuracy from variables for soil nutrients but also involves the prediction accuracy from spatial auto-correlation of soil nutrients. The BPNNOK was found to significantly improve the predictive accuracy between hyperspectral variables and soil nutrients. Furthermore, the BPNNOK model resolved the dependence on the density and uniform distribution of samples and enhanced spatial mapping performance, providing a new way to predict soil nutrients using hyperspectral remote sensing images as auxiliary variables.

## 5. Conclusions

In this study, hyperspectral remote sensing images were used as auxiliary variables and the spatial variability of soil nutrients was predicted using SLR, SVM, RF, BPNN and BPNNOK models. Compared with the SLR, SVM and RF models, the BPNN model with double hidden layers was more stable and had better fitting accuracy for soil TN (*RMSE* = 0.409 mg kg^−1^, *R*^2^ = 44.24%), soil AP (*RMSE* = 40.808 mg kg^−1^, *R*^2^ = 42.91%) and soil AK (*RMSE* = 67.464 mg kg^−1^, *R*^2^ = 48.53%). Furthermore, the BPNNOK model had the best fitting accuracy for soil TN (*RMSE* = 0.292 mg kg^−1^ and *R*^2^ = 68.51%), soil AP (*RMSE* = 29.62 mg kg^−1^ and *R*^2^ = 69.30%) and soil AK (*RMSE* = 49.67 mg kg^−1^ and *R*^2^ = 70.55%) compared to the other prediction models. The spatial mapping results of the BPNNOK model were closer to the real ranges of soil TN, soil AP and soil AK contents. Areas with high contents of soil TN, soil AP and soil AK in the study area were in the north-central, central and southwest and central and southeast regions, respectively. These findings indicate that the BPNNOK model demonstrated a better fitting ability for the nonlinear relationships between hyperspectral variables and soil nutrients. In addition, the most important bands (464–517 nm) for soil TN (b10, b14, b20 and b21), soil AP (b3, b19 and b22) and soil AK (b4, b11, b12 and b25) exhibited the best response and sensitivity. The application of hyperspectral VNIR bands (450–950 nm) allowed for rapid, efficient mapping and monitoring of soil nutrients at the regional scale. As shown in this study, the application of hyperspectral remote sensing image data and the BPNNOK model present potential ways to monitor soil nutrients and manage fertilization at the regional scale.

## Figures and Tables

**Table 1:** Descriptive statistics of soil nutrients (mg kg^−1^) in the study area.

	Data Set	Min	Max	Mean	SD	Skewness	Kurtosis	K-S	CV (%)
TN	Training set (n = 973)	0.12	3.04	1.18	0.53	0.223	−0.057	0.026	45.15
	Validation set (n = 324)	0.15	2.98	1.22	0.53	0.462	0.419	0.072	43.46
AP	Training set (n = 973)	2.2	261.6	75.54	53.84	1.033	0.507	0.000	71.28
	Validation set (n = 324)	2.4	257.5	74.81	53.52	1.08	0.657	0.000	71.54
AK	Training set (n = 973)	7	491	103.16	90.81	1.699	2.98	0.000	88.03
	Validation set (n = 324)	6	486	100.55	89.17	1.696	2.814	0.000	88.68

**Table 2:** Principal Component Analysis (PCA) of hyperspectral variables and their Pearson correlation analysis with soil nutrients.

	Principal Component Analysis			Pearson Correlation Analysis		
	Eigenvalue	Variance Explained (%)	Cumulative Value (%)	TN	AP	AK
PC1	19.36	62.45	62.45	**−0.56** ^1^	**0.131** ^2^	**0.126** ^2^
PC2	3.41	11.01	73.46	−0.15	**0.145** ^2^	**0.147** ^2^
PC3	2.71	8.73	82.19	**0.079** ^2^	−0.054	−0.053
PC4	1.48	4.77	86.96	**0.079** ^2^	−0.045	−0.023
PC5	1.08	3.50	90.46	0.023	−0.021	−0.035

^1^ Correlation is significant at *p* < 0.05 level; ^2^ Correlation is significant at *p* < 0.01 level.

**Table 3:** The prediction results of soil nutrients using Stepwise Linear Regression (SLR) and Support Vector Machine (SVM) models.

	Methods	*MAE* (mg kg^−1^)	*RMSE* (mg kg^−1^)	*R*^2^ (%)	*RPD*
TN	SLR	0.376	0.468	18.92	1.13
	SVM	0.372	0.448	27.00	1.19
AP	SLR	40.160	46.815	21.31	1.14
	SVM	39.599	45.634	26.44	1.17
AK	SLR	70.781	80.570	24.69	1.11
	SVM	67.732	77.903	29.52	1.14

**Table 4:** The prediction results of soil nutrients using Random Forest (RF) model.

		n_tree_^1^	node_size_^2^	tree_deep_^3^	*MAE* (mg kg^−1^)	*RMSE* (mg kg^−1^)	*R*^2^ (%)	*RPD*
RF	TN	1000	5	10	0.361	0.420	38.32	1.26
	AP				37.332	43.217	34.21	1.23
	AK				62.290	72.972	35.12	1.22

^1^ n_tree_: the number of trees are grown; ^2^ node_size_: the minimum size of the leaf; ^3^ tree_deep_: the maximum tree depth.

**Table 5:** The prediction results of soil nutrients using back-propagation neural network (BPNN) model.

		Architecture	*MAE* (mg kg^−1^)	*RMSE* (mg kg^−1^)	*R*^2^ (%)	*RPD*
BPNN	TN	5-25-20-1	0.328	0.409	44.24	1.30
	AP	5-20-15-1	35.554	40.808	42.91	1.31
	AK	5-20-15-1	59.434	67.464	48.53	1.32

**Table 6:** Semivariogram analysis of OK residuals of BPNN model for soil nutrients.

		Model	Range (km)	Nugget	Sill	Nugget Effect	*RMSE* (mg kg^−1^)
Residuals of BPNN	TN	Gaussian	3.524	0.173	0.007	0.961	0.453
	AP	Gaussian	19.069	1.639	0.054	0.968	45.91
	AK	Gaussian	2.84	3.764	0.587	0.865	79.56

**Table 7:** The predictive accuracy of soil nutrient contents using BPNNOK model at the validation set.

		*MAE* (mg kg^−1^)	*RMSE* (mg kg^−1^)	*R*^2^ (%)	*RPD*
BPNNOK	TN	0.213	0.292	68.51	1.82
	AP	21.22	29.62	69.30	1.81
	AK	33.10	49.67	70.55	1.80
